# Public Health Diplomas

**Published:** 1908-09-05

**Authors:** 


### PUBLIC HEALTH DIPLOMAS.

Diplomas in Public Health are granted by nearly all examining bodies. The Conjoint Board of England grants a diploma in public health. The Universities of Oxford, Cambridge, Durham, Liverpool, Manchester, Sheffield, and Leeds grant diplomas in hygiene and public health to their own graduates or to graduates of sister universities, and in some instances also grant diplomas to qualified practitioners who may not be graduates. The requirement for each diploma varies, but particulars may usually be obtained on application to the various registrars. The most popular diplomas appear to be the Cambridge D.P.H. and those granted by the University of Durham. For both the candidate must be a registered medical practitioner and must have attended special courses of instruction at recognised centres or with recognised teachers for a specified period. The Conjoint Board of Scotland requires candidates to produce evidence of attendance (subsequent to obtaining a registrable qualification) for six months at a Public Health Laboratory, and for six months under a Medical Officer of Health of a county or of a large urban district. There are two examinations, and candidates may present themselves for both of them at one period or for either examination separately. The fee is £12 12s., or £6 6s. in respect of each examination; and candidates referred are readmitted on a fee of £3 3s. in respect of each examination. The examination is held in Edinburgh or in Glasgow, there being two periods of examinations yearly—October and May. Applications for examination in Edinburgh to be sent to Mr. James Robertson, 54 George Square, Edinburgh; and in Glasgow to Mr. Alexander Duncan, B.A., LL.D., 242 St. Vincent Street, Glasgow, not later than fourteen days before the examination day. The University of Dublin confers degrees after examinations upon M.D.s or graduates in Medicine and Surgery of Dublin, Oxford, or Cambridge, and the Royal University only on graduates of Medicine of that University. The Conjoint Examining Board grants diplomas after examination to candidates who have complied with the regulations of the General Medical Council.

